# BCNU for recurrent glioblastoma multiforme: efficacy, toxicity and prognostic factors

**DOI:** 10.1186/1471-2407-10-30

**Published:** 2010-02-02

**Authors:** Thomas Reithmeier, Erika Graf, Tobias Piroth, Michael Trippel, Marcus O Pinsker, Guido Nikkhah

**Affiliations:** 1 Department of Stereotactic and Functional Neurosurgery, University Medical Center Freiburg, Breisacher Str. 64, 79106 Freiburg im Breisgau, Germany; 2 Clinical Trials Center, University Medical Center Freiburg, Germany, Breisacher Str. 64, 79106 Freiburg im Breisgau, Germany

## Abstract

**Background:**

The prognosis for patients with recurrent glioblastoma is still poor with a median survival between 3 and 6 months. Reports about the application of carmustine (BCNU), one of the standard chemotherapeutic drugs in the treatment of newly diagnosed glioblastoma, in the recurrent situation are rare.

**Methods:**

We performed a retrospective analysis of 35 patients with recurrent or progressive glioblastoma treated with 80 mg/m^2 ^BCNU on days 1 on 3 intravenously at our department for efficacy, toxicity and prognostic factors. Progression free survival and overall survival were estimated by the Kaplan-Meier method. The influence of age, Karnofsky performance status (KPS), tumor burden, pretreatment with temozolomide (TMZ), type of surgery for initial diagnosis and number of previous relapses on outcome was analyzed in a proportional hazards regression model.

**Results:**

The median age of the group was 53 years, median KPS was 70. Median progression free survival was 11 weeks (95% confidence interval [CI]: 8-15), median overall survival 22 weeks (95% CI: 18-27). The rate of adverse events, especially hematological toxicity, is relatively high, and in 3 patients treatment had to be terminated due to adverse events (one pulmonary embolism, one pulmonary fibrosis, and one severe bone marrow suppression). No influence of age, KPS, tumor burden, pre-treatment with TMZ and number of previous relapses on outcome could be demonstrated, while gross total resection prior to recurrence showed a borderline statistically significant negative impact on PFS and OS. These data compare well with historical survival figures. However prospective randomized studies are needed to evaluate BCNU efficacy against newer drugs like bevacizumab or the intensified temozolomide regime (one week on/one week off).

**Conclusion:**

In summary, BCNU treatment appears to be a valuable therapeutic option for recurrent glioblastomas, where no other validated radio- and/or chemotherapy are available.

## Background

Despite optimal treatment of patients with newly diagnosed glioblastoma multiforme by surgery, irradiation and chemotherapy, median survival is still only 14.6 months [1], and recurrent glioblastoma confers a dismal prognosis with a 6-months progression free survival (6M-PFS) rate of 15% to 21% and a median survival of 25 weeks [2]. This unfavorable prognosis is mainly due to the high propensity for tumor recurrence that inevitably occurs after a median survival time of 32 to 36 weeks [3,4]. The optimal treatment strategy in recurrent glioblastoma is ill-defined, and different chemotherapeutic regimes are used due to limited therapeutic options. Carmustine (BCNU) is one of the few chemotherapeutic drugs that are FDA approved for treatment of GBM according to positive experiences in the sixties with reported response rates of up to 30%. These report rates were mainly based on clinical criteria and might be overestimated. Few data exist about the efficacy of BCNU in patients with recurrent glioblastoma. Brandes [5] reported about 40 patients with recurrent glioblastoma treated with BCNU and found a six months progression free survival of 17.5% and a median time to progression of 13.3 weeks. However, all of these patients were chemo naive, and pretreatment consisted solely of surgery and irradiation. Since the study of Stupp [1] in 2005, the standard treatment for patients with primary glioblastoma includes a concomitant and adjuvant chemotherapy with temozolomide. As the cytotoxic effect of temozolomide and BCNU depends on its alkylating effect of the DNA, the considered resistance mechanisms of cancer cells could be equal effective for both drugs. In the case of temozolomide the temozolomide induced methyl adducts at the O^6^-guanine in DNA is repaired by the O^6^-methylguanine-DNA methyltransferase (MGMT) cytoprotective repair protein [6], and MGMT is also discussed as the main resistance mechanism against nitrosoureas [7]. However, no study investigated the impact of pretreatment with temozolomide on the efficacy of BCNU in the treatment of recurrent glioblastoma. Additionally the influence of tumor burden, age and Karnofsky performance status (KPS) and number of previous relapses on BCNU efficacy was also examined.

Therefore, this study was performed to obtain further data about the efficacy and toxicity of BCNU in recurrent glioblastoma that could serve as a benchmark for newer drugs, and to identify possible prognostic factors.

## Methods

### Patients' selection

We performed a retrospective review of the MS ACCESS database of our department for all patients with high-grade gliomas who received chemotherapy with BCNU between 12/2003 and 05/2008, and identified 93 patients. The patients' records as well as histological and radiological examinations were re-examined, and patients with a histologically proven glioblastoma, and radiological evidence of recurrent or progressive disease according to the Mac Donald criteria [8] or clinical progression were included into the study. A radiologically recurrent disease was defined as any new contrast enhancing area after complete resection of all contrast-enhancing tumor areas. A radiologically proven progressive disease was defined as any increase of contrast enhancing tumor area over 25% or an additional contrast enhancing area. Clinical progression was defined as the occurrence of significant neurological deterioration (e.g. disabling hemiparesis, aphasia, and deterioration of the general condition). Patients with a malignant transformation of a previous low-grade disease into a glioblastoma were also included. Patients in whom BCNU treatment of recurrent glioblastoma had been initiated before 2003 were excluded. According to these criteria, 35 of these 93 patients were eligible for this study. The study was approved by the local ethic committee.

### Treatment

The treatment schedule consisted of intravenous application of 80 mg/m^2 ^BCNU on days 1 to 3. The treatment cycle was repeated every 8 weeks for a maximum of 6 cycles. Patients were evaluated weekly for hematologic, renal, and hepatic toxicity. Treatment was terminated in the presence of either a neuroradiological or clinical progression or inacceptable toxicity (white blood cell count < 500 cells/μl; thrombocyte count < 10.000 cells/μl).

### Response evaluation

Patients underwent routinely neuroradiological and clinical evaluation for tumor response every 8 weeks or earlier, when clinical deterioration occurred. Radiological response was assessed according to the Mac Donald criteria [8]. A complete response was defined as the disappearance of all enhanced tumor, a partial response as a 50% or greater reduction in the largest cross-sectional tumor area and a progressive disease as a 25% increase in the size of the enhancing tumor or appearance of a new lesion. According to Norden et al. [9] we defined minimal response as a 25% to 49% reduction in the largest cross-sectional tumor area. All other situations were defined as radiologically stable disease. A clinical progression of the disease was defined as the occurrence of significant neurological deterioration (e.g. disabling hemiparesis, aphasia, and deterioration of the general condition). In case of a clinical or radiological progression BCNU treatment was terminated. In all other cases BCNU treatment was repeated up to a maximum of 6 cycles.

### Evaluation of tumor burden

The largest cross-sectional tumor area at start of the BCNU therapy was defined as the tumor burden and was the result of the multiplication of the largest cross-sectional diameters.

### Study endpoints

Study endpoints included evaluation of progression free and overall survival. Progression free survival was defined as the interval from start of BCNU treatment to radiological or clinical progression or death, whichever occurred first. Overall survival was defined as the time span from start of BCNU until death from any cause. Patients' general practitioners were contacted between May and September 2008 to obtain the most recent follow-up information.

### Statistical methods

Progression free and overall survival were estimated by the Kaplan-Meier method, censoring observations at the time of last follow-up if the respective event was not observed. Median follow-up was derived from the estimated censoring distribution. Prognostic factors (age, KPS, tumor burden, gross total resection, pretreatment with TMZ, number of previous relapses) were all fitted together in an exploratory fashion into a Cox regression model, both as continuous and dichotomised predictors. Two-sided 95% confidence intervals were used and statistical significance defined as p < 0.05 based on two-sided tests.

## Results

### Patients' characteristics

Thirty-five patients were included into the study. 34 patients had a histologically proven GBM, in one patient diagnosis of GBM was confirmed in the combination of histopathological, neuroradiological and clinical findings. There were 21 (60%) men and 14 (40%) women with a median age of 53 years (range: 27 to 77 years). 26 patients (74%) were older than 45 years and median KPS at start of BCNU treatment was 70 (range: 40-90), 37% of the patients had a KPS below 70. 	Tumor involved one lobe in 22 patients (12 temporal, 5 frontal, 4 parietal, 1 central), two or more lobes in 10 patients (6 parieto-temporal, 2 fronto-temporal, 1 temporo-parietal, 1 fronto-temporo-parietal) and was multifocal in 3 patients. The operative procedure at time of first diagnosis of GBM was a gross total resection in 15, a partial resection in 4 and a stereotactic biopsy in 16 patients. 31 (89%) patients received postoperative radiotherapy to limited fields and 24 (69%) additionally an adjuvant and concomitant chemotherapy with temozolomide. In 30 patients BCNU therapy was initiated after the first relapse, in four patients after the second relapse (first relapse was treated in 2 patients with stereotactical application of an immunotoxin, in 1 patients with an alternative chemotherapeutic regime (cilengitide) and in one patients a circumscribed tumor progress was irradiated with LINAC) and in one patient after the fourth relapse (first relapse treated with LINAC, second and third relapse operated). Median time span from initial diagnosis to start of BCNU therapy was 38 weeks (range: 11-189 weeks). For further details see also table 1 and 2.

**Table 1:** Patients' characteristics

Sex			
	Male	21	(60%)
	Female	14	(40%)
			
Age, years			

	Median	53	
	Range	27-77	
	< = 45	9	(26%)
	> 45	27	(74%)
			
Tumor burden (product of largest cross-sectional diameters), mm2			

	Median	736	
	Range	20-3519	
	Not measurable	n = 8	(23%)
			
KPS			

	Median	70	
	Range	40-90	
	90	7	(20%)
	70-80	15	(43%)
	<70	13	(37%)
			
Time from diagnosis to start of BCNU, weeks			

	Median	38 weeks	
	Range	11-189 weeks	
			
Primary therapy			

	Total resection	15	(43%)
	Partial resection	4	(11%)
	Biopsy	16	(46%)
	Temozolomide	24	(71%)
	Radiation	31	(89%)
			
Number of relapses at start of BCNU therapy			

	First	30	(86%)
	Second	4	(11%)
	Third	0	(0%)
	Fourth	1	(3%)

**Table 2:** Treatment modalities in the relapse situation

	First relapse	Second relapse	Third relapse	Fourth relapse
BCNU	30	4	0	1
Resection	0	1	1	0
LINAC radiosurgery	2	0	0	0
Local immunotoxin administration	2	0	0	0

Alternative chemotherapy (cilengitide)	1	0	0	0

### Toxicity

62 cycles of BCNU were administered, ranging from 1 to 4 cycles, with a mean of 1.8 cycles per patient. Bone marrow toxicity, coagulopathies, pulmonal and infectious complications were evaluated according to the common toxicity criteria for adverse events V3.0. In summary 7 patients developed one or more adverse events during BCNU therapy. 4 patients developed thrombocytopenia grade 3 or 4, 5 patients developed leucopenia grade 3 or 4 and 1 patient developed an anemia grade 3. Two patients developed infection grade 3 or 4 and one patients a thromboembolic event grade 4 associated with pulmonary embolism. One patient experienced an interstitial lung fibrosis grade 3 after the administration of the third cycle BCNU. All patients with bone marrow toxicities grade III or IV have been pretreated with temozolomide, have been administered two or more cycles of BCNU and have had a stereotactic biopsy. In three patients BCNU treatment was terminated due to adverse events (one patient with pulmonary embolism, one patient with pulmonary fibrosis, one patient with extensive myelotoxicity).

### Response rates

In two patients a partial response and in 19 patients a stable disease was observed. We found no patients with a minimal or complete response. 11 patients showed a progressive disease after application of the first cycle. In three patients evaluation of radiological response was not possible.

### Progression free and overall survival

**Figure 1:**
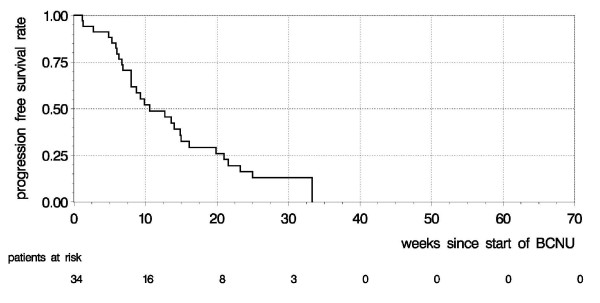
**Progression free survival**. Kaplan-Meier curve showing progression free survival for recurrent glioblastoma multiforme after BCNU chemotherapy.

**Figure 2:**
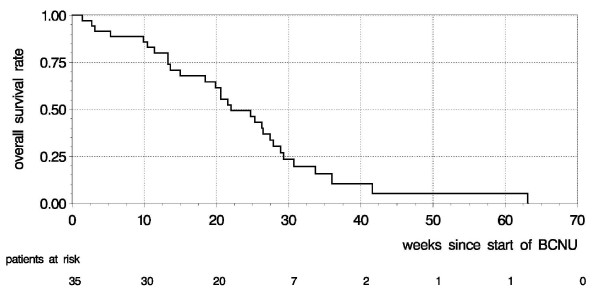
**Overall survival**. Kaplan-Meier curve showing overall survival for recurrent glioblastoma multiforme after BCNU chemotherapy.

Of 35 patients, 29 progressed within the follow-up period, 5 were alive and progression-free, and one patient died 28 weeks after initiation of BCNU with no data on progression available (omitted from analysis of progression free survival). 30 patients died, 5 were alive at the end of follow-up. Median progression free survival was 11 weeks (95% confidence interval [CI]: 8-15 weeks), with a 6 months progression free survival rate of 13% (Figure 1; median follow-up: 30 months). Median overall survival was 22 weeks (95% CI: 18-27 weeks), 43% survived for more than 6 months (Figure 2).

In an explorative proportional hazards regression model for the effects of dichotomized prognostic factors (age, KPS, tumor burden, gross total resection prior to recurrence, pre-treatment with TMZ and number of previous relapses) on progression free survival, only gross total resection showed a borderline statistically significant association with a higher risk of relapse (hazard ratio of progression 3.11 for gross total resection compared with partial resection/biopsy; 95% CI 1.02-9.52; p = 0.047; table 3). The same model was used for overall survival and also identified only gross total resection as a statistically significant negative prognostic factor for survival (hazard ratio of overall survival 3.034 for complete resection compared with partial resection/biopsy; 95% CI 1.05-8.7; p = 0.04; table 4).

**Table 3:** Determinants of progression-free survival after BCNU treatment of recurrent glioblastoma (n = 34, 30 events)

		Hazard ratio*	95% confidence interval	p-value
Age (years)	≤ 53 vs. > 53 (the median)	1.12	0.41-2.98	0.844

Tumor size (mm^2^)	≤ 736 vs. > 736 (the median)	0.46	0.15-1.42	0.177
	not measurable vs. > 736	0.45	0.16-1.28	0.135

Karnofsky performance score	≤ 70 vs. 80 or 90	0.50	0.20-1.26	0.143

Surgery	Complete resection vs. biopsy/partial resection	3.11	1.02-9.52	0.047

Pre-treatment with temozolomide	yes vs. no	1.30	0.43-3.91	0.641

Previous relapses	1 vs. more than 1	0.48	0.14-1.61	0.232

* A hazard ratio <1 (>1) indicates an effect in favor of the first (the second) group in terms of progression-free survival.

**Table 4:** Determinants of overall survival after BCNU treatment of recurrent glioblastoma (n = 34, 30 events)

		Hazard ratio*	95% confidence interval	p-value
Age (years)	≤ 53 vs. > 53 (the median)	0.56	0.22-1.45	0.233

Tumor size (mm^2^)	≤ 736 vs. > 736 (the median)	0.61	0.22-1.71	0.349
	not measurable vs. > 736	0.55	0.18-1.66	0.287

Karnofsky performance score	≤ 70 vs. 80 or 90	1.24	0.50-3.07	0.648

Surgery	Complete resection vs. biopsy/partial resection	3.03	1.05-8.74	0.040

Pre-treatment with temozolomide	yes vs. no	0.81	0.31-2.13	0.674

Previous relapses	1 vs. more than 1	1.81	0.51-6.48	0.357

*A hazard ratio <1 (>1) indicates an effect in favor of the first (the second) group in terms of progression-free survival.

## Discussion

The natural history and optimal treatment of recurrent glioblastoma is not well-defined due to lack of uniform definition and criteria for tumor recurrence, institutional variability in treatment strategy and the heterogeneous nature of the disease including location of recurrence. Treatment options for recurrent glioblastoma include surgical intervention, chemotherapy or irradiation (stereotactic radiosurgery and brachytherapy). The rationale for reoperation is to reduce intracranial pressure, improve neurological status of the patient and possibly improve efficacy of adjunctive therapy. Additionally it offers the possibility of BCNU-impregnated wafer insertion into the resection cavity and in combination with irinotecan median survival rates of 13.5 months are reported [10]. Overall, resection may provide a modest benefit in survival and/or improvement in quality of life within a subset of patients and should only be considered in patients with a KPS score > 70 with lesions in favourable locations [1,11,12].

Stereotactic radiosurgery (SRS) is a treatment option for small recurrent glioblastoma. Median survival of patients undergoing single-fraction SRS is around 10 months [13-15] and can be applied as an outpatient therapy. Brachytherapy has a similar efficacy as SRS, with a median survival time of 9.1 months [16] and a 3-year survival rate of 15% [17], but only 20-30% of patients with recurrent glioblastoma meet the morphological and focal criteria for application of interstitial brachytherapy.

For these reasons chemotherapy is still the most common treatment option for recurrent glioblastoma multiforme [18]. Besides the use of temozolomide, carboplatin, procarbazine and imatinib mesylate, which are hypothesized to reduce the patient's risk of death by approximately 15% [19], are the most widely used drugs. Wong et al. [2] summarized the results of different phase II studies for recurrent high-grade gliomas and found 6 months progression free survival rate of 15% for patients with recurrent glioblastomas. Overall survival was 30 weeks, however with no differentiation between anaplastic astrocytoma and glioblastoma. Wick et al. [20] suggested an intensified TMZ scheme (one week on/one week off) and found 6-months-PFS of 48% in a patient population with a favourable KPS. Vredenburgh et al. [21] applied a combined therapy of bevacizumab and irinotecan in 35 patients and reported 6-months-PFS of 46% and an overall survival of 42 weeks.

However, the role of BCNU - one of the few drugs with proven activity against GBM - in this situation is poorly defined. Brandes et al. [22] performed the only phase II trial with BCNU in recurrent glioblastoma and reported a median time to progression of 13.3 weeks and 6 months PFS rate of 17.5%.

Our results compare well with the reported 6-months progression free survival and overall survival rates reported by Brandes [22] and Wong [2] but more recently introduced drugs like bevacizumab or intensified TMZ scheme (one week on/one week off) seems to further improve 6 months PFS and overall survival. However, the limited effect of BCNU on PFS in our series can in part be explained by the problem of pseudoprogression in malignant glioma. This phenomenon describes progressive and enhancing lesions on MRI, which are not related to tumor progression, but which are treatment effects [23] and has been originally described after chemoradiotherapy with temozolomide. As in 16 patients in our series BCNU therapy was terminated due to a radiographic progress of the disease without any clinical deterioration it can be assumed that these changes might be in some cases treatment related and not a true objective tumor progression. However pseudoprogression after BCNU therapy has not been described in the literature to date and therefore we would suggest to perform a radiological follow-up examination in patients with radiological signs of tumor progression to rule out pseudoprogression and a misleadingly termination of therapy.

It has to be considered that our study population was very heterogeneous with a high variation of tumor burden and a high proportion of patients older than 45 years, who had already received various therapeutic modalities and were not only treated for the first but also for a second or fourth relapse of the disease. Therefore, we investigated if age, KPS, tumor burden, number of previous relapses or pre-treatment with TMZ had any impact of patient's PFS or overall survival.

As TMZ and BCNU are counteracted via the same resistance mechanism, we hypothesized that pre-treatment with TMZ could have led to a resistance against alkylating agents reducing the efficacy of BCNU. We found no statistically significant impact of TMZ pre-treatment on PFS or overall survival, so that BCNU is still a therapeutic option for patients progressive under TMZ as the role of alkylating agents in the development of chemotherapeutic alkylating drug resistance remains still unclear [24]. However, one limitation of this study is the missing information about the status of MGMT promoter methylation. Molecular studies have demonstrated that the benefit from alklyating chemotherapy is mainly observed in patients with a methylated MGMT gene promoter, and are thus unable to repair some of the chemotherapy-induced DNA damage [25,26] and MGMT expressing tumor cells are 4- to 10-fold more resistant to BCNU, temozolomide and related compounds [27,28]. Molecular and epigenetic characterization of gliomas is essential in future glioma therapy to develop individual therapeutic strategies and to overcome the MGMT mediated chemoresistance. One concept is to irreversibly inactivate MGMT through administration of O^6^-Benzylguanine, a compound that reacts with MGMT by covalent transfer of the benzyl group to the active site-cysteine and renders by this way the tumor cells 2- to 14-fold more sensitive to alkylating agents in vitro and in vivo settings [29] and phase II trials to prove the potential benefit of a combination of O^6^-Benzylguanine with temozolomide or BCNU are currently under way [24].

The tumor burden at the beginning of BCNU therapy had no influence on PFS or overall survival. This is in line with the level II recommendations for the surgical management of newly diagnosed glioblastomas, that recommend that only maximal cytoreductive surgery (>98% of tumor) correlates with a significant effect on survival [30-32]. Therefore, reoperation for recurrent GBM should only be considered when a maximally safe and maximally cytoreductive surgery is possible.

Brandes [5], who also investigated the influence of age, KPS and response to chemotherapy on prognosis, found that on multivariate analysis, only response to chemotherapy remained an independent prognostic factor for time to progression.

In summary, we could not identify an influence of age, KPS, tumor burden, pre-treatment with TMZ or number of previous relapses on PFS or OS in our patient population. The data do suggest a negative impact of a gross total resection prior to recurrence in terms of PFS and OS, This might be due to a negative selection of patients with extensive tumor growth after initial complete resection who are not amenable for reoperation.

## Conclusion

Based on these findings we would recommend for patients with recurrent glioblastoma, who are not enrolled into clinical studies, to start with the intensified temozolomide scheme due to less toxic effects and superior progression free survival figures compared to BCNU. However, as we found no objective prognostic factors of BCNU efficacy it represents a valuable therapeutic option after a further progression when no other validated treatment modalities are available and the high rate of toxicity, especially after pre-treamtent with temozolomide has to be considered. Prospective and randomized studies are needed to compare the efficacy of nitroso-ureas against newer drugs like bevacizumab to prove their therapeutic superiority in the recidive situation.

## Competing interests

The authors declare that they have no competing interests.

## Authors' contributions

TR was responsible for the conception and design of the study; TR and TP performed the collection and assembly of data; EG, TR, TP, MP, MT, GN performed the statistical analysis and interpretation of data; TR, EG and GN wrote the manuscript; TR, EG, TP, MT, MP and GN revised the manuscript. All authors read and approved the final manuscript.

## Pre-publication history

The pre-publication history for this paper can be accessed here:

http://www.biomedcentral.com/1471-2407/10/30/prepub
